# Analysis of Direction-Finding Performance of Vector Hydrophones Based on Unmanned Underwater Vehicle Platforms and Application Research of Embodied Cognition Theory

**DOI:** 10.3390/s25237239

**Published:** 2025-11-27

**Authors:** Hu Zhang, Honggang Zhang, Linsen Zhang, Bo Tang

**Affiliations:** Naval University of Engineering, Wuhan 430033, China; 2120213033@nue.edu.cn (H.Z.); 1212061017@nue.edu.cn (L.Z.); 1312021011@nue.edu.cn (B.T.)

**Keywords:** unmanned underwater vehicle, vector hydrophone, embodied cognition, direction-finding performance, scattered sound field, embodied transfer function

## Abstract

To address the problem of platform scattering interference in direction finding using vector hydrophones mounted on unmanned underwater vehicle (UUV) platforms, this paper introduces a direction-finding error compensation method based on embodied transfer function (ETF) correction within the framework of embodied cognition theory. By establishing an analytical model of the scattered sound field of an infinite rigid cylinder, the influence mechanism of the UUV platform on the sound pressure and vibration velocity measurements of the vector hydrophone is systematically investigated, and the concepts of sound pressure ETF and vibration velocity ETF are defined. The research results indicate that at an operating frequency of 800 Hz, the ETF-based direction-finding method reduces the average direction-finding error from 8.8° to 6.2°, representing a performance improvement of 30.2%. Moreover, when the target lies near the transverse, the direction-finding error of the embodied model remains below 1.5°. This study provides novel theoretical support and a technical framework for achieving high-precision direction finding of vector hydrophones mounted on UUV platforms.

## 1. Introduction

Unmanned underwater vehicles are emerging as a popular research topic for underwater target detection equipment. Currently, the detection sonars carried by UUVs are primarily side-array and towed-array systems [1,2,3]. As a sensor capable of simultaneously measuring underwater sound pressure and vibration velocity, the vector hydrophone offers advantages such as compact size, low weight, and high sensitivity. It enables underwater target localization by calculating sound intensity across azimuths through combined sound pressure–vibration velocity signal processing, thus showing broad application potential in underwater unmanned platforms with limited space [4,5,6,7,8,9].

In recent years, research institutions in various countries have advanced the integration of vector hydrophones with unmanned platforms. For example, the Massachusetts Institute of Technology in the United States installed a 100 m-long vector hydrophone towed array on the “Bluefin-21” UUV, enabling the detection and tracking of ship targets [10]. The University of Pisa in Italy, in collaboration with the Italian Navy and the NATO Centre for Maritime Research and Experimentation, suspended a single vector hydrophone 1.5 m below the UUV using an elastic rope, achieving real-time passive detection and azimuth estimation of 1 kHz line-spectrum targets with a direction-finding error within 2° [11,12]. The Portuguese Naval Research Center, in cooperation with several universities, permanently mounted a dual-accelerometer vector hydrophone 0.5 m beneath a UUV, successfully achieving target localization with a maximum bearing deviation of 10° [13,14,15].

Beyond configurations where the vector hydrophone is suspended below the UUV, it can also be fabricated into a conformal structure using MEMS technology and embedded within the UUV surface [16,17]. Another design involves mounting the hydrophone on the top of an underwater glider, yielding a direction-finding error of approximately 5° [18]; when multiple layers of sound-absorbing materials are applied to the glider top, the direction-finding error of a vector hydrophone positioned 0.4 m above it can be constrained within 5° [19].

In underwater target localization using vector hydrophones, in addition to the sound intensity method, each hydrophone channel can be regarded as a multi-element array. By exploiting the inherent array manifold characteristics of a single vector hydrophone, conventional array signal processing algorithms can be applied to it [8,20,21,22,23]. When operated independently, both classes of direction-finding algorithms can accurately estimate target bearings, achieving theoretical errors below 1° [24,25]. However, when the vector hydrophone is installed on a UUV platform, the direction-finding error increases substantially, and the underlying mechanism of this error generation has not yet been systematically analyzed.

Previous studies have confirmed that vector hydrophones mounted on UUV platforms can effectively achieve long-range, real-time autonomous detection of underwater and surface targets. Nevertheless, the installation of a vector hydrophone on a UUV causes scattering of the incident acoustic wave by the UUV hull, such that the received signal comprises both the incident and platform-scattered components, deviating from the free-field condition. Even when sound-absorbing materials are applied to the UUV surface, direction-finding deviations persist, and these deviations are closely related to parameters including UUV geometry, dimensions, signal frequency, and the hydrophone’s distance from the platform.

Recently, advances in embodied cognition theory within the sonar detection domain have provided a novel approach to addressing this issue. The theory posits that intelligent agents enhance perceptual capability through dynamic interaction between the “body” (i.e., the sonar platform) and the environment, establishing a closed-loop learning paradigm driven jointly by physical modeling and data. This framework offers a promising pathway for mitigating platform scattering interference [26].

Accordingly, this study, grounded in embodied cognition theory, proposes a comprehensive sound pressure–vibration velocity embodied transfer function (ETF) correction method. The mechanism by which UUV platform scattering affects the direction-finding performance of vector hydrophones is systematically examined, and the effectiveness of the proposed approach is validated through simulation analyses. Compared to other advanced approaches, including deep learning methods [27,28] and improvements to traditional signal processing techniques [29,30], the embodied transfer function systematically treats scattering effects as an integral component of the body (i.e., the UUV platform) perception system, rather than as interference sources requiring elimination through statistical learning or algorithmic optimization. This body–environment interaction optimization model for perception demonstrates certain advantages in small-sample learning, environmental adaptability, and interpretability.

## 2. Direction-Finding Error Analysis Model of Vector Hydrophones Based on UUV Platform

### 2.1. Traditional UUV Scattered Sound Field Model

When the underwater target signal propagates toward the UUV, the platform scatters the incident acoustic wave. Consequently, the signal received by the vector hydrophone in the vicinity of the UUV is no longer the direct incident wave, but a composite signal formed by the superposition of the incident and scattered components. This phenomenon introduces errors in the target localization of the vector hydrophone. To investigate the mechanism underlying this error, the most effective approach is to analyze the characteristics of the sound field once the signal impinges upon the UUV.

Since the main bodies of UUV platforms are predominantly cylindrical, scattering from the regular cylindrical surface at the mid-section constitutes the primary scattering source. Scattering from the end faces is typically confined to a very narrow angular range near the bow and stern directions, and the geometric surface area is also relatively small. This means that cylindrical surface scattering is the dominant factor across most azimuth angles. Therefore, under the assumption of neglecting end-face scattering effects [31,32], the acoustic field at the mid-section of a finite-length cylindrical hull (excluding both ends) can be approximated as that of an infinitely long rigid cylinder. The classical separation-of-variables method can then be employed to solve for the scattered acoustic field [33]. However, this simplification has practical limitations. Although significantly smaller compared to scattering in the transverse direction, reflections and diffraction along the UUV’s fore-and-aft axis do alter the acoustic field. However, this assumption captures the most fundamental and universal physical characteristics of the scattering field, representing a reasonable compromise between theoretical depth and engineering complexity.

**Figure 1:**
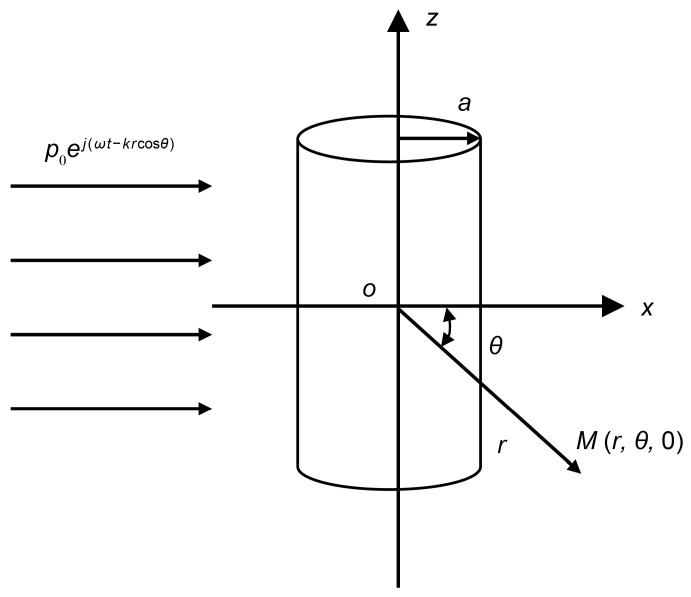
Model of a plane wave incident on a cylinder.

Based on this assumption, this paper considers an infinite rigid cylinder as the research object and establishes the analytical model illustrated in Figure 1.

An infinite rigid cylinder is positioned at the origin of a cylindrical coordinate system, and a plane acoustic wave is incident along the *x*-axis. The radius of the cylinder is denoted as *a*. When the plane wave encounters the cylinder, scattering occurs due to the rigid boundary condition. Point M is the position coordinate of any point on the tangent plane in the circumferential direction that forms an angle *θ* with the *x*-axis and is situated at a distance r from the coordinate origin. The expression for the sound pressure of the incident wave is given as follows:

(1) $$p_{i}=p_{0}e^{j(\omega t-kx)}=p_{0}e^{j(\omega t-kr\cos\theta )}.$$

In the formula, *k* denotes the wave number, *λ* represents the wavelength, *c* is the sound speed in the water medium, and *ω* is the angular frequency. By decomposing the incident plane wave into a superposition of cylindrical waves, the decomposition expression for the incident plane wave can be derived as follows [34]:

(2) $$p_{i}(r,\theta )=p_{0}e^{-jkr\cos\theta }=\left[J_{0}(kr)+2\sum_{n=1}^{\infty }{\left(-j\right)}^{n}J_{n}(kr)\cos(n\theta )\right]p_{0}.$$

When the amplitude on the cylindrical wavefront is uniformly distributed in all directions, *n* = 0, and the incident plane wave can be simplified as follows:

(3) $$p_{i}(r,\theta )=p_{0}\sum_{n=0}^{\infty }\varepsilon_{n}{\left(-j\right)}^{n}J_{n}(kr)\cos(n\theta ),\;\varepsilon_{n}=\left\{\begin{array}{l} 1,\;n=0\\ 2,\;n=1,\;2,\;3\ldots \end{array}\right..$$

Based on the Euler equation, the expressions for the radial particle vibration velocity and the axial particle vibration velocity of the incident wave can be derived as follows:

(4) $$\left\{\begin{array}{l} u_{ir}=-\frac{p_{0}}{j\rho c}\left[-J_{1}(kr)+2\sum_{n=1}^{\infty }{\left(-j\right)}^{n}\frac{dJ_{n}(kr)}{d(kr)}\cos(n\theta )\right]\\ u_{i\theta }=\frac{p_{0}}{j\omega \rho r}2\sum_{n=1}^{\infty }{\left(-j\right)}^{n}J_{n}(kr)n\sin(n\theta ) \end{array}\right..$$

In the formula, *ρ* denotes the density of the water medium. As the rigid cylinder is positioned within the sound field, its surface and the resulting scattered waves can be considered as a superposition of cylindrical waves of various orders. Therefore, the sound pressure of the scattered wave, *p_s_*, can be expressed as follows:

(5) $$p_{s}(r,\theta ,t)=\sum_{n=0}^{\infty }A_{n}H_{n}^{\left(2\right)}(kr)\cdot \cos(n\theta ).$$

In the above formula, *H_n_*^(2)^(*kr*) represents the Hankel function of the second kind, *A_n_* is the corresponding coefficient, and its value is determined by the boundary conditions. Both the incident wave and the scattered wave must satisfy the condition that the radial particle vibration velocity on the rigid boundary equals zero, that is:

(6) $${\left.\left(u_{in}+u_{sn}\right)\right|}_{r=a}=0,\;i.e.,\;{\left.\frac{\partial }{\partial r}(p_{i}+p_{s})\right|}_{r=a}=0,$$

By substituting Equations (2) and (5) into the boundary condition (6), the following expression can be obtained:

(7) $$\sum_{m=0}^{\infty }A_{m}{\left.\frac{\partial H_{m}^{\left(2\right)}(kr)}{\partial r}\right|}_{r=a}\cos(m\theta )=-p_{0}\sum_{m=0}^{\infty }{\left(-j\right)}^{m}\varepsilon_{m}{\left.\frac{\partial J_{m}(kr)}{\partial r}\right|}_{r=a}\cos(m\theta ).$$

By utilizing the orthogonality and completeness of the $\left\{\cos(m\theta )\right\}$ , the expression for the scattered sound pressure of the infinite rigid cylinder can be derived as follows:

(8) $$p_{s}(r,\theta ,t)=p_{0}\sum_{n=0}^{\infty }b_{n}H_{n}^{\left(2\right)}(kr)\cos(m\theta ).$$

In the formula, the coefficient *b_n_* is expressed as follows:

(9) $$b_{n}=-\frac{{\left(-j\right)}^{n}\varepsilon_{n}{\left.\frac{\partial J_{n}(kr)}{\partial r}\right|}_{r=a}}{{\left.\frac{\partial H_{n}^{\left(2\right)}(kr)}{\partial r}\right|}_{r=a}}.$$

Similarly, the radial particle vibration velocity $u_{sr}$ and the axial particle vibration velocity $u_{s\theta }$ in the scattered acoustic wave field can be obtained from the Euler equation, respectively, as follows:

(10) $$\left\{\begin{array}{l} u_{sr}=-\frac{p_{o}}{j\rho c}\sum_{n=0}^{\infty }b_{n}\frac{dH_{n}^{\left(2\right)}(kr)}{d(kr)}\cos(n\theta )\\ u_{s\theta }=\frac{p_{o}}{j\omega \rho r}\sum_{n=0}^{\infty }b_{n}H_{n}^{\left(2\right)}(kr)n\sin(n\theta ) \end{array}\right..$$

Therefore, the expressions for the sound pressure and particle vibration velocity in the total sound field are given as follows:

(11) $$p_{total}=p_{i}+p_{s}=p_{0}\sum_{n=0}^{\infty }\left[\varepsilon_{n}{\left(-j\right)}^{n}J_{n}(kr)\cdot \cos(n\theta )+b_{n}H_{n}^{\left(2\right)}(kr)\cdot \cos(n\theta )\right],$$

(12) $$\begin{array}{l} u_{total\_r}=u_{ir}+u_{sr}\\ =-\frac{p_{0}}{j\rho c}\left[-J_{1}(kr)+2\sum_{n=1}^{\infty }{\left(-j\right)}^{n}\frac{dJ_{n}(kr)}{d(kr)}\cos(n\theta )-\sum_{n=0}^{\infty }b_{n}\frac{dH_{n}^{\left(2\right)}(kr)}{d(kr)}\cos(n\theta )\right] \end{array},$$

(13) $$\begin{array}{l} u_{total\_\theta }=u_{i\theta }+u_{s\theta }\\ =\frac{p_{0}}{j\omega \rho r}\left[2\sum_{n=1}^{\infty }{\left(-j\right)}^{n}J_{n}(kr)n\sin(n\theta )-\sum_{n=0}^{\infty }b_{n}H_{n}^{\left(2\right)}(kr)n\sin(n\theta )\right] \end{array}.$$

In the Cartesian coordinate system, the components of the vibration velocity can be expressed as follows:

(14) $$u_{x}=u_{total\_r}\cdot \cos\theta -u_{total\_\theta }\cdot \sin\theta ,$$

(15) $$u_{y}=u_{total\_r}\cdot \sin\theta +u_{total\_\theta }\cdot \cos\theta .$$

The above analysis demonstrates that after the acoustic wave is incident on the UUV, the expressions for sound pressure and vibration velocity in the total sound field—including the scattered components—can be explicitly obtained. This provides a theoretical basis for analyzing the scattering sound field characteristics of the UUV, investigating the direction-finding errors of vector hydrophones, and constructing the ETF.

### 2.2. Construction of the UUV Platform Embodied Cognition Model

According to embodied cognition theory, the emergence and evolution of intelligence depend on specific bodily structures and activities [35]. In sonar detection, the shell of the sonar platform represents the body, whose acoustic characteristics should not be treated as sources of interference but rather as exploitable resources for enhancing perceptual capability [26]. For a UUV platform, its acoustic properties are determined by factors such as material composition, structural configuration, and motion state. To quantitatively describe the modulation effect of the body on the received signal, the sound pressure ETF $U_{ETF}^{p}$ and the vibration velocity ETF $U_{ETF}^{v}$ are defined, with their frequency-domain expressions given as follows:

(16) $$U_{ETF}^{p}\left(r,a,\theta ,f\right)=\frac{P_{emb}\left(r,a,\theta ,f\right)}{P_{0}\left(f\right)},$$

(17) $$U_{ETF}^{v}\left(r,a,\theta ,f\right)=\frac{V_{emb}\left(r,a,\theta ,f\right)}{V_{0}\left(f\right)}.$$

In the formula, $P_{emb}$ and $V_{emb}$ represent the total sound pressure and total vibration velocity received by the vector hydrophone under the embodied model, respectively; *P*_0_ and *V*_0_ denote the free-field sound pressure and free-field vibration velocity at the reference point (the UUV centroid), respectively. Here, *r* is the installation distance between the vector hydrophone and the UUV surface, *a* is the UUV radius, *θ* is the target azimuth, and *f* is the signal frequency.

Based on the embodied model, the sound pressure and vibration velocity signals received by the vector hydrophone mounted on the UUV platform can be expressed as follows:

(18) $$p_{emb}=U_{ETF}^{p}\cdot p_{i}\cdot e^{j\phi_{m}}+n_{p},$$

(19) $$u_{emb}=U_{ETF}^{v}\cdot u_{i}\cdot e^{j\phi_{m}}+n_{u}.$$

In the formula, $\phi_{m}=k\cdot \Delta r_{m}$ represents the phase compensation term introduced by the path difference caused by the UUV platform; $n_{p}$ and $n_{u}$ denote the noise signals in the sound pressure and vibration velocity reception processes, respectively.

It can be observed from Equations (16) and (17) that the ETF reflects the influence of three key parameters—frequency, installation distance, and target azimuth—on the scattering effect. Its output value (i.e., its degree of deviation from 1) directly reflects the intensity of the body–environment interaction, providing a basis for correction in direction-finding algorithms. This interaction manifests in the core variables of the ETF. Here, the frequency *f* determines the wave number *k*, while *ka* is the key dimensionless parameter describing the matching degree between body dimensions and environmental acoustic wave wavelengths. When *ka* ≪ 1 (i.e., low frequencies, where the wavelength far exceeds the UUV dimensions), environmental sound waves predominantly diffract around the body with minimal scattering. At this point, the output value is approximately equal to 1, indicating negligible interference from the body on the environmental sound field. When *ka* ≈ 1 (i.e., mid-frequencies, where the wavelength is comparable to UUV dimensions), environmental sound waves resonate with the body, causing scattering. The deviation from 1 is maximal, reflecting the body’s significant influence on the environmental sound field. When *ka* ≫ 1 (i.e., high frequencies, where the wavelength is much smaller than the UUV dimensions), geometric reflection of the ambient acoustic field dominates, leading to a stable scattering-correction value.

Similarly, the installation distance *r*—the perceived position on the body—determines the interference weight of the body’s scattered field on the incident sound field, reflected in the variable *kr*. When *r* → 0 (near field), the hydrophone is adjacent to the body, where the scattered field dominates, causing the output value to deviate most significantly from 1. When *r* → ∞ (far field), the hydrophone is distant from the body, the scattered field attenuates, and the output value gradually approaches 1. This indicates that the body’s interference with the ambient sound field becomes negligible, returning to a free-field state. The target azimuth angle *θ* (the direction of incident sound waves) determines the relative orientation between the body and the sound waves. This orientation difference is reflected in the cos *θ* and sin *θ* terms within the equation.

**Figure 2:**
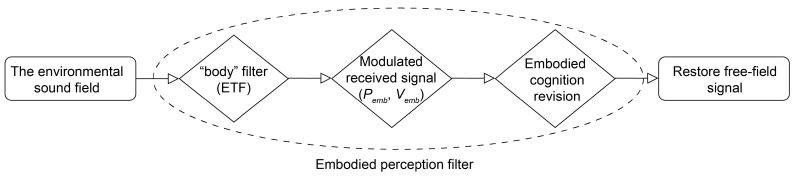
Embodied perception filtering.

The nonlinear relationship between the aforementioned core variables and the ETF systematically and quantitatively describes how the body alters and shapes the perceptual inputs (sound pressure and vibroacoustic velocity) of the agent (the UUV underwater perception system). By systematically correcting the received signal using the sound pressure ETF $U_{ETF}^{p}$ and the vibration-velocity ETF $U_{ETF}^{v}$ , as shown in Equations (18) and (19), a “body-aware model” was established, achieving embodied-perception filtering functionality, as illustrated in Figure 2. To further elucidate its physical principles, it is necessary to analyze the scattered acoustic field of the UUV platform and the characteristics of the EFT model.

## 3. Simulation of UUV Platform Scattered Sound Field and Analysis of ETF Characteristics

When the vector hydrophone is mounted on the exterior of the cylindrical main body of the UUV, the *x*-channel is aligned with the radial direction of the cylinder (for measuring the radial vibration velocity component), while the *y*-channel is parallel to the cylinder’s axial direction (for measuring the axial vibration velocity component). Consequently, analyzing variations in the sound pressure field, radial vibration velocity field, and axial vibration velocity field enables an accurate reflection of the received signal characteristics of each vector hydrophone channel. These variations are strongly influenced by the signal wavelength, UUV radius, and the spacing between the vector hydrophone and the UUV.

### 3.1. Scattered Sound Field at Different Frequencies

**Figure 3:**
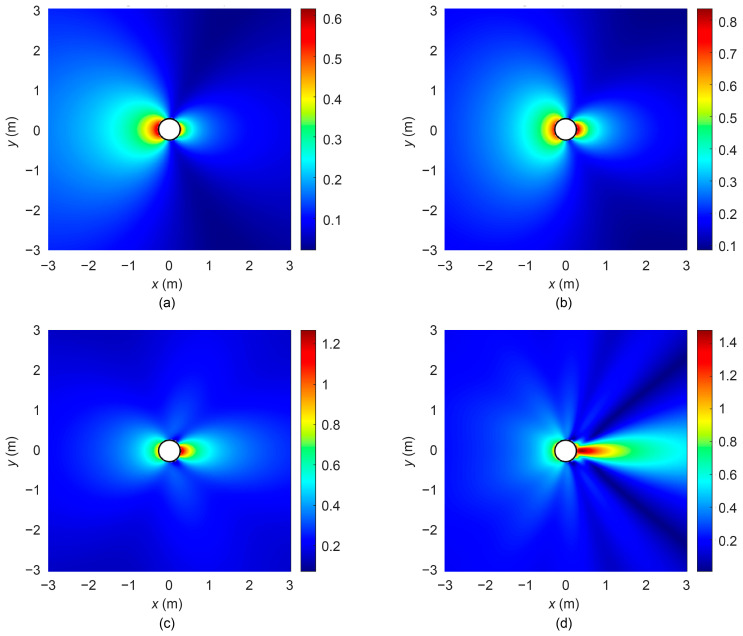
Scattered sound pressure at different frequencies. (**a**) λ=4πa ; (**b**) λ=2πa ; (**c**) λ=πa ; (**d**) λ=πa/3 .

**Figure 4:**
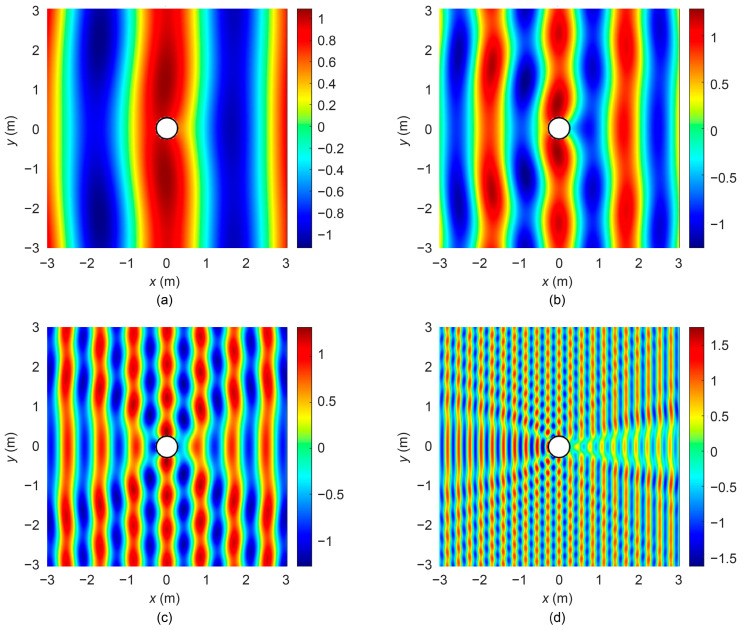
Total sound pressure in the sound field at different frequencies. (**a**) λ=4πa ; (**b**) λ=2πa ; (**c**) λ=πa ; (**d**) λ=πa/3 .

Typically, the diameter of a medium-sized UUV is 533 mm; thus, in this study, the cylinder radius *a* is set to 0.267 m. When plane waves of varying frequencies were incident along the radial (*x*-axis) direction of the cylinder, the distributions of the scattered sound pressure and total sound pressure were obtained using Equations (8) and (11), as illustrated in Figure 3 and Figure 4.

**Figure 5:**
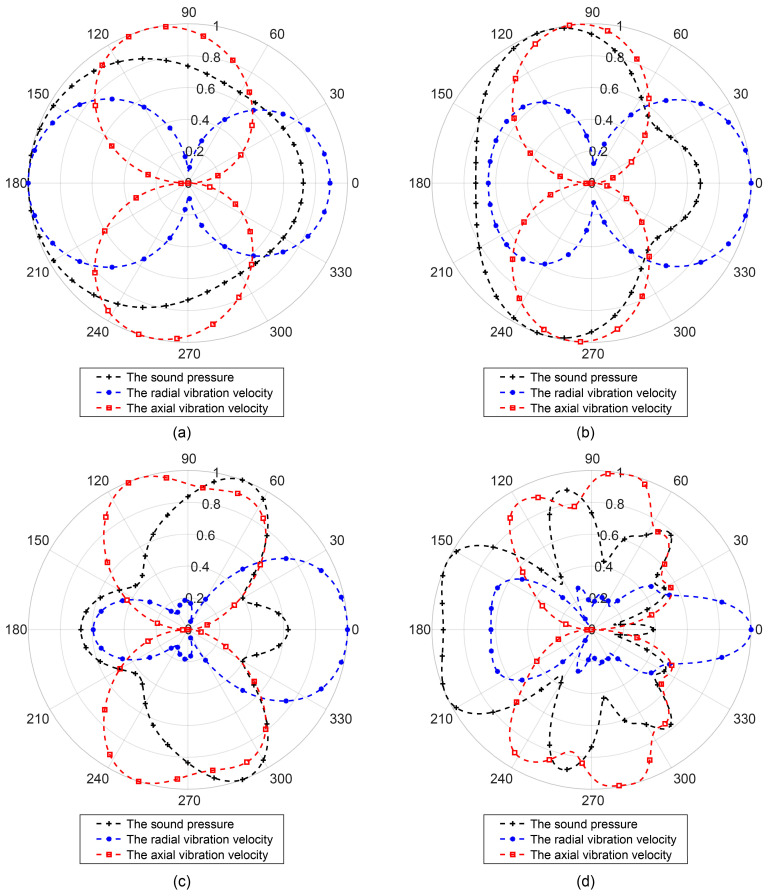
Directivity diagrams of the sound field at different frequencies. (**a**) λ=4πa ; (**b**) λ=2πa ; (**c**) λ=πa ; (**d**) λ=πa/3 .

Figure 3 and Figure 4 illustrate the distributions of the scattered sound pressure and total sound pressure at different frequencies. It can be observed from the figures that at low frequencies, the wavelength is much larger than the cylinder dimensions, resulting in a minor influence of scattered waves on the sound field. As the frequency increases, the wavelength gradually approaches the cylinder size, and diffraction effects become evident. A scattered wave with strong directivity forms behind the cylinder, and the higher the frequency, the narrower the beam width. The scattering in front of the cylinder (facing the direction of incident wave propagation) remains more uniform than that behind the cylinder, a phenomenon that can be further verified from the directivity diagrams in Figure 5.

Figure 5 presents the directivity diagrams of sound pressure and vibration velocity in the sound field at different frequencies, measured at a distance of 0.3 m from the cylinder surface. The results indicate that at lower frequencies, the influence of cylinder scattering on the sound field is minimal, the spatial distribution of sound pressure is nearly uniform, and its directivity diagram approximates a circle, while the vibration velocity directivity diagram exhibits an “8”-shaped pattern. As the frequency increases ( λ<2πa ), the sound pressure in front of the cylinder interferes with the incident wave due to the scattered field, leading to the appearance of concave regions in the directivity pattern. Behind the cylinder, diffraction generates side lobes in the directivity diagram, and the number of side lobes increases with frequency.

In summary, when λ≥2πa , the influence of the UUV cylindrical platform on the sound field remains relatively small. However, as the frequency rises and the wavelength becomes comparable to the platform size, the scattered wave effects become increasingly pronounced, causing notable distortion in the directivity patterns of both sound pressure and vibration velocity.

### 3.2. Spatial Distribution of Sound Field at Different Installation Distances of the Vector Hydrophone

As shown in Figure 3 and Figure 4, the sound field distribution varied with distance around the cylinder. Therefore, it is essential to analyze the spatial distribution characteristics of the sound field at different installation distances to determine the optimal position of the vector hydrophone—namely, the position where the UUV has the least influence on the sound field.

**Figure 6:**
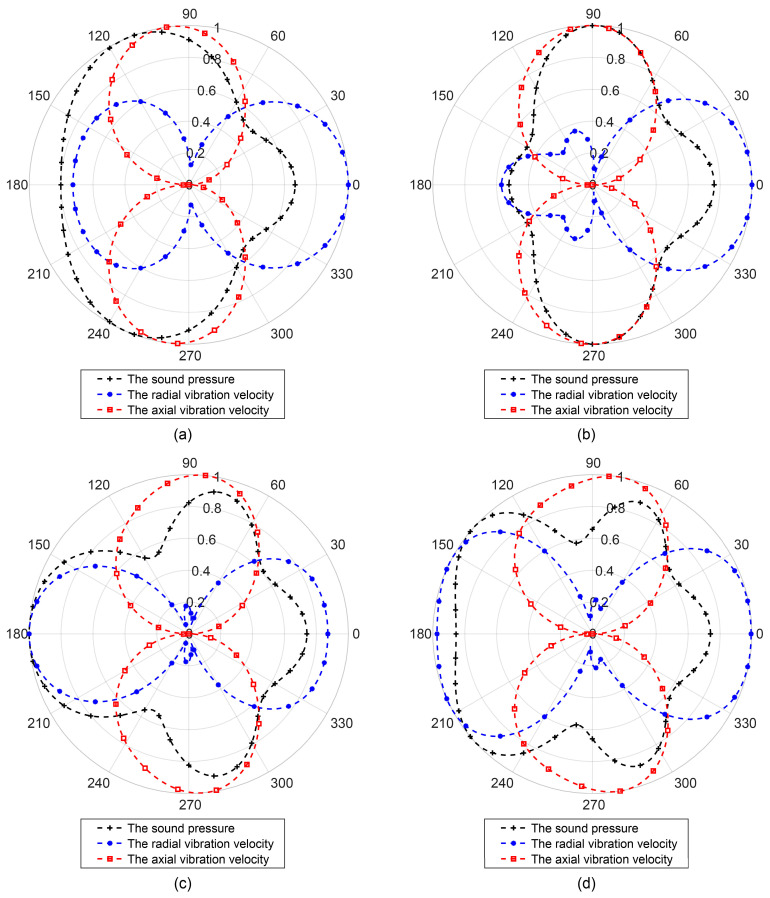
Directivity diagrams of the sound field at different installation distances ( λ=2πa ). (**a**) *r* = *a*; (**b**) *r* = 2*a*; (**c**) *r* = 3*a*; (**d**) *r* = 4*a*.

Let λ=2πa , and place the vector hydrophone at distances *r* = *a*, *r* = 2*a*, *r* = 3*a*, and *r* = 4*a* from the cylinder surface. The corresponding sound field directivity patterns are illustrated in Figure 6.

Figure 6 demonstrates that the sound field distribution differs at various positions. Overall, the sound pressure distribution in front of the cylinder (within the azimuth range of 130–230°) is more uniform than that behind it. The sound pressure behind the cylinder exhibits strong fluctuations with increasing distance. The radial vibration velocity directivity diagram displays a pronounced tapered shape at *r* = 2*a*, while the axial vibration velocity directivity remains relatively stable, maintaining a clear “8”-shaped pattern. As the distance increases further, multiple main lobes emerge in the sound pressure field, gradually forming a circular pattern. This indicates that as the vector hydrophone moves farther from the cylinder, the influence of scattered waves diminishes, and the directivity of both sound pressure and particle vibration velocity approaches that of the free-field condition.

In conclusion, a larger UUV platform size corresponds to a lower upper operating frequency limit for the vector hydrophone it carries. When the wavelength becomes comparable to the platform size, scattered waves cause significant distortion of the sound field. Additionally, when the vector hydrophone is positioned in front of the cylinder (facing the incident wave direction), the influence of platform scattering is smaller than that behind the cylinder. As the installation distance increases, the effect of scattered-wave interference gradually weakens. Therefore, appropriately selecting the installation distance can improve the direction-finding performance of the vector hydrophone for underwater targets.

### 3.3. Analysis of ETF Characteristics

**Figure 7:**
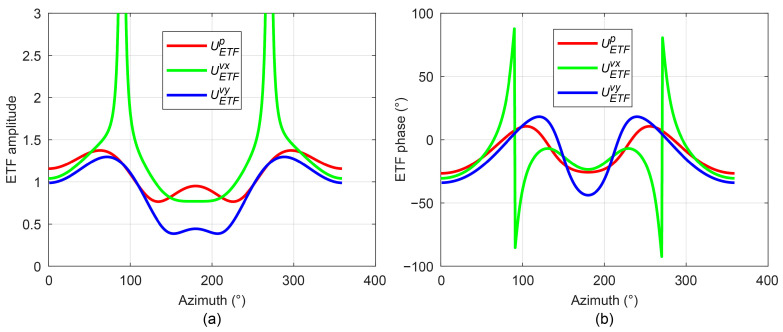
Characteristics of the embodied transfer function. (**a**) Amplitude characteristics; (**b**) Phase characteristics.

To analyze the characteristics of the ETF and verify its capability to compensate for direction-finding errors, this study employs the analytical solution of the cylindrical scattered sound field developed in Section 2.1 to establish the ETFs $U_{ETF}^{p}$ , $U_{ETF}^{v_{x}}$ , and $U_{ETF}^{v_{y}}$ for sound pressure and the vibration velocity components in the *x* and *y* directions, respectively. The simulation parameters are set as follows: UUV radius *a* = 0.267 m, hydrophone installation distance *r* = 0.567 m, frequency *f* = 800 Hz, and sound speed *c* = 1500 m/s. The numerically calculated ETF characteristics are presented in Figure 7.

The simulation results indicate that, due to UUV platform scattering, the sound pressure ETF exhibits maximum values near 60° and 290°, and minimum values near 130° and 220°. The *y*-direction vibration velocity ETF follows a trend similar to that of the sound pressure ETF but demonstrates phase differences at specific azimuths. In contrast, the *x*-direction vibration velocity ETF shows distinct behavior, with characteristic extrema occurring around 90° and 270°, accompanied by phase inversion.

## 4. Direction-Finding Algorithms and Performance Analysis

The vector hydrophone can determine the sound intensity in each channel by combining the sound pressure and vibration velocity information from multiple channels, thereby estimating the target azimuth through the ratio of sound intensities across channels [36,37]. Among the available methods, the cross-spectrum method is one of the most widely used direction-finding techniques for vector hydrophones. It estimates both the horizontal azimuth and the elevation angle by analyzing the cross-power spectral density relationship between sound pressure and vibration velocity.

### 4.1. Traditional Cross-Spectrum Direction-Finding Method

Assume that the sound pressure of the incident acoustic wave is *p_s_*, and the corresponding vibration velocity is *v_s_*. The total signal received by the vector hydrophone can then be expressed as follows:

(20) $$\left\{\begin{array}{l} p=p_{s}+p_{n}\\ v=v_{s}+v_{n} \end{array}\right..$$

In the formula, *p_n_* and *v_n_* represent the noise signals associated with the sound pressure and vibration velocity, respectively. By correlating *p_s_* with *p*, and *p_s_* with *v*, the corresponding correlation functions can be obtained as follows:

(21) $$\left\{\begin{array}{l} R_{pp_{s}}(\tau )=E\left\{p_{s}(t)\cdot p^{*}(t+\tau )\right\}=E\left\{p_{s}(t)\cdot p_{s}^{*}(t+\tau )\right\}+E\left\{p_{s}(t)\cdot p_{n}^{*}(t+\tau )\right\}\\ R_{p_{s}v}(\tau )=E\left\{p_{s}(t)\cdot v^{*}(t+\tau )\right\}=E\left\{p_{s}(t)\cdot v_{s}^{*}(t+\tau )\right\}+E\left\{p_{s}(t)\cdot v_{n}^{*}(t+\tau )\right\} \end{array}\right..$$

Since *p_s_* is independent of *p_n_* and *p_s_* is also independent of *v_n_*, Equation (21) can be simplified as follows:

(22) $$\left\{\begin{array}{l} R_{pp_{s}}(\tau )=E\left\{p_{s}(t)\cdot {p_{s}}^{*}(t+\tau )\right\}\\ R_{p_{s}v}(\tau )=E\left\{p_{s}(t)\cdot {v_{s}}^{*}(t+\tau )\right\} \end{array}\right..$$

Then, the cross-correlation functions between *p_s_* and the three orthogonal components of the vibration velocity, *v_x_*, *vᵧ*, and *v_z_*, are expressed as follows:

(23) $$\left\{\begin{array}{l} R_{p_{s}v_{x}}(\tau )=E\left\{p_{s}(t)\cdot {v_{sx}}^{*}(t+\tau )\right\}\\ R_{p_{s}v_{y}}(\tau )=E\left\{p_{s}(t)\cdot {v_{sy}}^{*}(t+\tau )\right\}\\ R_{p_{s}v_{z}}(\tau )=E\left\{p_{s}(t)\cdot {v_{sz}}^{*}(t+\tau )\right\} \end{array}\right..$$

In the formula, *E*{·} denotes the correlation function operator, and “*” represents the complex conjugate operator. By performing a Fourier transform on the above correlation functions, the corresponding power spectrum functions can be obtained as follows:

(24) $$\left\{\begin{array}{l} S_{p_{s}v_{x}}(f)=\int_{-\infty }^{\infty }R_{p_{s}v_{x}}(\tau )e^{-j2\pi f\tau }d\tau \\ S_{p_{s}v_{y}}(f)=\int_{-\infty }^{\infty }R_{p_{s}v_{y}}(\tau )e^{-j2\pi f\tau }d\tau \\ S_{p_{s}v_{z}}(f)=\int_{-\infty }^{\infty }R_{p_{s}v_{z}}(\tau )e^{-j2\pi f\tau }d\tau \end{array}\right..$$

In the above expressions, $S_{p_{s}v_{x}}\left(f\right)$ , $S_{p_{s}v_{y}}\left(f\right)$ , and $S_{p_{s}v_{z}}\left(f\right)$ represent the cross-spectral density functions between the sound pressure signal received by the vector hydrophone and the vibration velocity components *v_x_*, *v_y_*, and *v_z_*, respectively. According to the sound intensity–angle relationship, the direction of arrival can be estimated using Equation (25). The horizontal azimuth *θ* and elevation *α* are:

(25) $$\left\{\begin{array}{l} \alpha =\tan^{-1}\left[\frac{\sqrt{S_{p_{s}v_{x}}^{2}(f)+S_{p_{s}v_{y}}^{2}(f)}}{S_{p_{s}v_{z}}(f)}\right]\\ \theta =\tan^{-1}\left[\frac{S_{p_{s}v_{y}}(f)}{S_{p_{s}v_{x}}(f)}\right] \end{array}\right..$$

Since the cross-spectrum function derived from Equation (25) may contain an imaginary component, and the useful signal information resides solely in the real part, it is sufficient to analyze only the real part to obtain the horizontal azimuth and elevation angle, which are expressed as follows:

(26) α=tan−1ReSpsvx2(f)+ReSpsvy2(f)ReSpsvz(f)θ=tan−1ReSpsvy(f)ReSpsvx(f).

### 4.2. Embodied Cognition Direction-Finding Method

Based on the embodied model signal, the traditional cross-spectrum method is refined to account for the influence of the UUV platform, and its sound pressure–vibration velocity cross-spectral density is expressed as follows:

(27) $$\begin{array}{l} S_{p,v}^{emb}=E\left[P_{emb}\cdot V_{emb}^{*}\right]\\ \;\;=U_{ETF}^{p}\cdot U_{ETF}^{v\;*}\cdot S_{p_{0},v_{0}}\cdot e^{j2\phi_{m}}+S_{n_{p},n_{v}} \end{array}.$$

In the formula, $S_{p_{0},v_{0}}=E\left[P_{0}\cdot V_{0}^{*}\right]$ represents the free-field cross-spectrum, and $S_{n_{p},n_{v}}$ denotes the noise cross-spectrum. Under these conditions, the target azimuth can be expressed as follows:

(28) θ=argReSp,vemb⋅UETFp−1⋅UETFv⋅e−j2ϕm.

Compared with the traditional cross-spectrum method, the improved algorithm compensates for signal distortion induced by UUV scattering through the ETF and corrects the phase deviation resulting from the path difference introduced by the UUV platform through $\phi_{m}$ . In Equations (26) and (28), Re represents the real portion.

### 4.3. Comparative Analysis of Direction-Finding Performance

**Figure 8:**
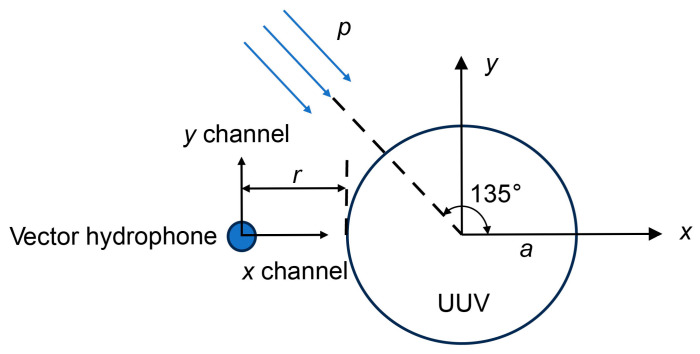
Schematic diagram of the underwater target azimuth.

To verify the enhancement achieved by the direction-finding method based on the ETF in improving the accuracy of vector hydrophones under UUV platform scattering, a simulation model was established, as shown in Figure 8. The simulation parameters are set as follows: UUV radius *a* = 0.267 m; the vector hydrophone is positioned directly in front of the cylinder at a distance *r* = 0.3 m from the cylinder surface; the *x*-channel is aligned with the *x*-axis direction, the *y*-channel is parallel to the *y*-axis direction, and the underwater target is located at the 135° azimuth relative to the UUV.

**Figure 9:**
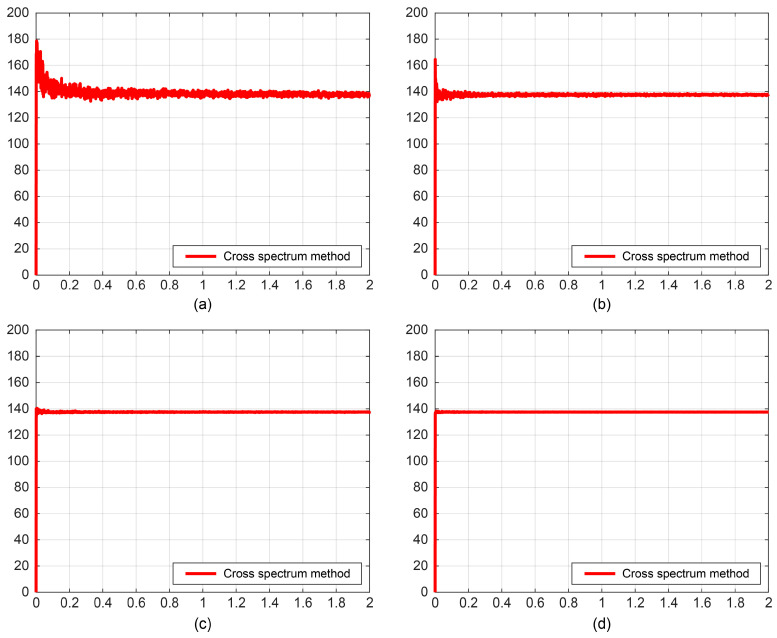
Direction-finding results under different signal-to-noise ratios. (**a**) SNR = −15 dB; (**b**) SNR = −5 dB; (**c**) SNR = 5 dB; (**d**) SNR = 15 dB.

The target is assumed to emit a single-frequency signal with *f* = 800 Hz. The received signal sampling frequency is *f_s_* = 8 kHz, the water medium density *ρ* = 1000 kg/m^3^, the sound speed *c* = 1500 m/s, the sampling time *t* = 2 s, and the number of sampling points $N=f_{s}\cdot t$ . First, the target azimuth estimated by the traditional direction-finding method at different signal-to-noise ratios (SNRs) is computed, as shown in Figure 9.

As illustrated in Figure 9, during the 0–2 s signal acquisition period, the bearing estimation gradually stabilizes as the time gain increases. Furthermore, the higher the SNR, the shorter the time required for the faster stabilization of the bearing results. Specifically, at SNR = −15 dB, the bearing error is approximately 7°; when the SNR exceeds 5 dB, the bearing error remains within 5°.

**Figure 10:**
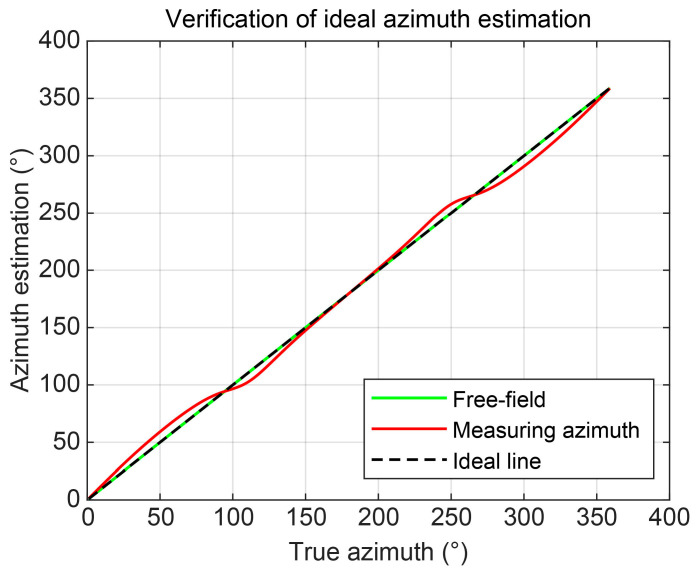
Variation in measured azimuth angle with true azimuth of the target.

Similarly, at other azimuths, the bearing measurement results are also affected by platform scattering, as shown in Figure 10. The results indicate a monotonically increasing relationship between the measured and true azimuths. Considering the characteristics of the scattered acoustic field, when the target is positioned on the same side as the vector hydrophone (within the 130–230° range), the bearing error remains within 5°. This phenomenon reflects the “embodied” characteristics of the platform.

Table 1 presents the averaged results of multiple calculations for several typical azimuths using both direction-finding methods under an SNR of 5 dB.

**Figure 11:**
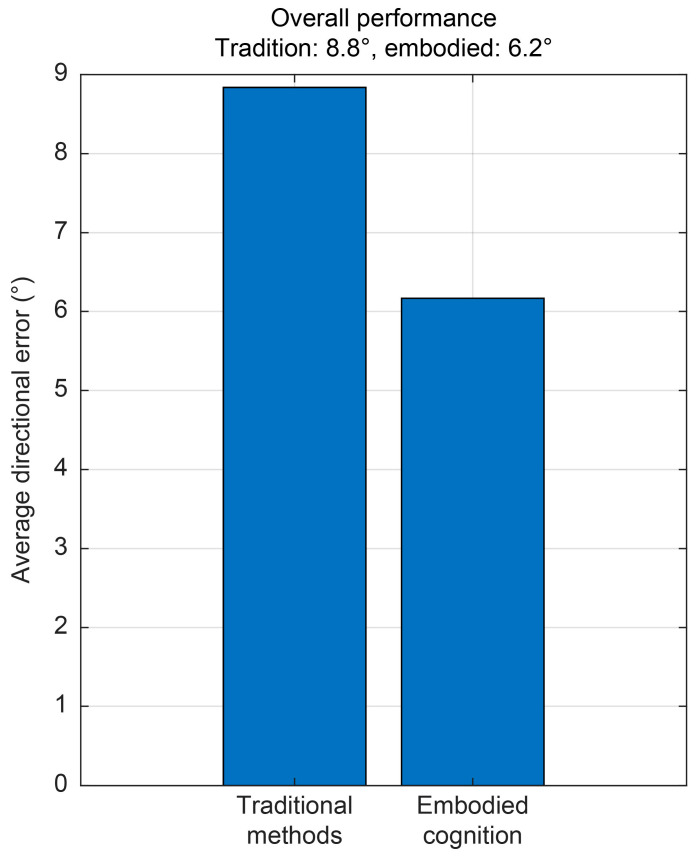
Overall performance comparison between the two direction-finding methods.

As shown in Table 1, within the 135–170° azimuth range, the bearing error of the traditional method remains within 5°, while the ETF–corrected method further reduces it to within 1.5°. In addition, the ETF-corrected approach shows improved accuracy across other azimuth regions. Overall statistical results indicate that the traditional direction-finding method has an average error of 8.84°, whereas the embodied cognition-based method achieves an average error of 6.17°, corresponding to a 30.2% performance improvement, as illustrated in Figure 11.

As indicated by the preceding analysis, the direction-finding results are influenced not only by changes in the target’s bearing but also by the frequency, a characteristic inherent to the body. Table 2 and Table 3 present the comparison results at 500 Hz and 1200 Hz under a signal-to-noise ratio of 5 dB.

By comparing the data in Table 1, Table 2 and Table 3, it can be seen that at different frequencies, the measurement errors of both methods remain within 5° near the 0° and 180° azimuths. After correction by the ETF, the directional performance improved by 30.6% at 500 Hz and by 6.8% at 1200 Hz. The improvements in efficiency are more noticeable at lower frequencies, implying that the ETF is frequency-dependent. However, at various frequencies, significant errors remain at specific azimuth angles. Possible reasons for this include:

1. The complex interference patterns formed by the incident and scattered waves

Since the incident wave and scattered wave are functions of frequency, distance, and azimuth angle, the total sound field resulting from their superposition under different conditions exhibits differing spatial distribution characteristics. This leads to the formation of ‘nodes’ or ‘anti-nodes’ in certain azimuthal directions where the scattered field and incident field interfere. As a result, the amplitude ratio and phase difference between sound pressure and particle velocity undergo drastic changes, disrupting the linear mapping relationship between the sound intensity vector and azimuth.

2. High-order modal truncation error:

When calculating the scattered field at specified angles (e.g., 60° and 120°), some higher-order modes of rigid cylinder scattering may be triggered. These higher-order modes are truncated in the series summation (i.e., only a finite number of terms are taken), thereby introducing model error.

3. The embodied transfer function exhibits strong correlations with frequency and direction:

Although the embodied cognition approach, modified by the ETF, partially compensates for distortion effects and restores the correct mapping relationship between sound intensity vectors and azimuth, the ETF also exhibits strong dependence on frequency and azimuth, as platform scattering is intrinsically linked to the frequency—an inherent characteristic of the body. From this, it is evident that the initial ETF correction is insufficient to fully restore the free field.

4. Distortion of the phase relationship between sound pressure and particle velocity:

In the total sound field formed by the interference of the incident field and the scattered field, variations in the particle velocity field are more complex than those in the sound pressure field, which is reflected in the pattern of the sound field’s directivity. Vector hydrophones measure both sound pressure and particle velocity simultaneously, and DOA estimation is performed through combined processing of these signals. The relevant algorithms require a specific phase relationship between sound pressure and particle velocity. However, in strongly interfering regions at certain angles, the phase relationship becomes highly linear. Consequently, simple frequency-domain division (i.e., ETF filtering) is insufficient to fully correct these distortions.

## 5. Conclusions and Outlook

This study investigated the direction-finding performance of vector hydrophones on UUV platforms, explored their application methods, and examined both the acoustic field characteristics of UUVs and the direction-finding performance of vector hydrophones for underwater targets. The present study introduced the embodied cognition theory in direction-finding research involving vector hydrophones mounted on UUV platforms. A comprehensive theoretical framework for the ETF of sound pressure and particle velocity was proposed.

First, based on a rigid infinite-cylinder model, theoretical expressions for sound pressure and particle velocity in the scattered acoustic field were derived. Subsequently, simulation analyses were performed to investigate the scattered acoustic field characteristics of the UUV platform, focusing on frequency-dependent behavior and directional distributions of sound pressure and vibration velocity at different installation distances. Furthermore, an embodied cognition model for UUV-mounted vector hydrophones was established, defining a coupled sound pressure–velocity ETF to quantify the interaction between the body and the surrounding acoustic field. The traditional cross-spectrum direction-finding algorithm was then refined to incorporate ETF correction, addressing a core limitation of conventional models that overlook platform scattering.

The study revealed that a larger UUV platform corresponded to a lower upper operating frequency limit for the mounted vector hydrophone. When the acoustic wavelength became comparable to the platform size, scattered waves induced pronounced distortion in the acoustic field. Moreover, as the installation distance of the vector hydrophone from the UUV increases, the influence of platform-induced scattering decreases. The embodied perception direction-finding method, corrected using the ETF, reduces the average direction-finding error from 8.8° to 6.2° at an operating frequency of 800 Hz, thus achieving a 30.2% improvement in performance. Additionally, the analysis identifies two primary error mechanisms—amplitude distortion and phase distortion—arising from platform scattering. When the target lies near the transverse, the embodied model constrains the bearing error within 1.5°, thereby supporting efficient detection by vector hydrophones on UUV platforms and facilitating compact UUV configurations suitable for close-proximity installations.

## Figures and Tables

**Table 1:** Directional performance comparison at 800 Hz (unit: °).

Target Azimuth	Traditional Methods (Average Error)	Embodied Cognition Method (Average Error)
15°	4.8	1.8
45°	12.6	7.2
60°	16.1	14.9
120°	18.4	15.8
135°	4.9	1.0
150°	3.4	1.3
170°	1.7	1.1

**Table 2:** Directional performance comparison at 500 Hz (unit: °).

Target Azimuth	Traditional Methods (Average Error)	Embodied Cognition Method (Average Error)
15°	3.2	1.2
45°	7.6	3.0
60°	13.3	11.9
120°	13.9	12.5
135°	7.2	4.3
150°	4.4	1.4
170°	2.9	1.2

**Table 3:** Directional performance comparison at 1200 Hz (unit: °).

Target Azimuth	Traditional Methods (Average Error)	Embodied Cognition Method (Average Error)
15°	7.2	4.8
45°	14.9	14.3
60°	16.2	15.4
120°	7.9	6.9
135°	11.4	10.3
150°	9.1	8.0
170°	2.5	1.2

## Data Availability

The original contributions presented in this study are included in the article. Further inquiries can be directed to the corresponding author.

## Abbreviations

The following abbreviations are used in this manuscript:

ETF	Embodied transfer function
SNRs	Signal-to-noise ratios
UUV	Unmanned underwater vehicle
